# Editorial: The Gastrointestinal Fate and Health Effects of Dietary Antioxidants

**DOI:** 10.3389/fnut.2022.915283

**Published:** 2022-05-23

**Authors:** Guadalupe F. Loarca-Piña, Gustavo A. González-Aguilar, Abraham Wall-Medrano

**Affiliations:** ^1^Programa de Posgrado en Alimentos del Centro de la República (PROPAC), Research and Graduate Students in Food Science, School of Chemistry, Universidad Autónoma de Queretaro, Querétaro, Mexico; ^2^Laboratorio de Antioxidantes y Alimentos Funcionales, Coordinación de Tecnología de Alimentos de Origen Vegetal, Centro de Investigación en Alimentacion y Desarrollo, Hermosillo, Mexico; ^3^Coordinación de Ciencias de la Salud, Instituto de Ciencias Biomédicas, Universidad Autónoma de Ciudad Juárez, Ciudad Juárez, Mexico

**Keywords:** antioxidants, biotransformation, metabolomics, *in silico* analysis, nutraceutical, digestibility, medicinal chemistry

All mammal cells possess a well-orchestrated redox system tightly connected with other metabolic processes. Many metabolic intermediaries are timely fabricated or destroyed based on their oxidation state and the type and amount of intracellular free radicals regulate the expression of multiple proteins, affecting cell metabolism and survival in a concentration-dependent manner. Also, antioxidant nutrients (e.g., vitamins, minerals, and peptides) and xenobiotics (e.g., phenolic compounds, saponins), play a major role in reducing the odds of cellular oxidative stress, by exerting direct (e.g., radical scavenging), and indirect (e.g., epigenetic action) mechanisms (1). Many chronic gastrointestinal (GI) and systemic illnesses are related to unbalanced redox homeostasis that can be counteracted with dietary antioxidants; basic/applied scientific research in this field, has backed up the developing “antioxidant” segment within a billion USD functional food and or nutraceutical market, a market segment particularly boosted during COVID-19 pandemic (2).

The dose-dependent cellular effect of any dietary antioxidant is partially affected by its gastrointestinal fate and first/second-pass metabolism. Dietary antioxidants are released from food matrices (bioaccessibility), selectively absorbed by the GI epithelia, and/or bio-transformed (parent compounds-to-metabolites) by the enteric host's and microbiota's biochemical machinery in a random order, to improve their bioavailability (3). As soon as these phytochemicals or its metabolites enter systemic circulation, they are subject to first and second-pass hepatic metabolism, before reaching target cells (including hepatocytes) to exert their bioactivity. The antioxidant/epigenetic effect of a given parent/metabolite antioxidant can be reduced or enhanced during this process. It is noteworthy that much of the accumulated knowledge on the metabolomic fate of dietary antioxidants comes from well-executed metabolomic platforms that combine high-trough output analytical methods and bioinformatic tools, setting a new way of evaluating the pharmacodynamical action of dietary antioxidants (4, 5). This Research Topic of *Frontiers in Nutrition* attempted to gather basic scientific knowledge on the health-promoting effects of food-derived antioxidants and over-the-counter (OTC)-nutraceuticals, with particular emphasis on the structure-function processes associated with their intraluminal bioaccessibility and bioavailability as well as their absorption, distribution, and metabolism (target organs), excretion, and toxicity (ADMET).

In functional dietetics, prescribing the best natural sources or designer foods to guarantee the maximum enteral bioaccessibility/absorption and systemic bioavailability of food-derived antioxidants, is a common practice. Such recommendation initially comes from population-based nutrition-health surveys that often associate consuming certain food items with a particular disease-prevention or disease-ameliorating effect. In their review article, Elizalde-Romero et al. provide enough evidence on the richness, structural diversity, gastrointestinal stability, systemic fate, and main health-promoting effects of many phytochemicals present in Solanaceae edible vegetables (fruit, roots, and aerealparts). The authors succinctly state that the health effects of vegetables such as tomato (*S. lycopersicum*), black nightshade (*S. migrum*), potato (*S. tuberosum*), and eggplant (*S. melongena*), are associated with their natural richness in antioxidants (polyphenols, carotenoids) and non-antioxidant (alkaloids, saponins) phytochemicals but whose activity is partially conditioned by their structure-specific bioaccessibility and food processing/enteral stability.

The health benefits of pulses have been documented in many epidemiological studies and human clinical trials while certain systematic/narrative reviews have postulated that certain health benefits are varietal (genotype/phenotype)-specific (6). Méndez-López et al. reviewed and documented (*in silico*) the immunity-promoting activity of certain phytochemicals (flavonoids, chalcones, stilbenoids, jasmonates, alkaloids, and L-DOPA) and dietary fiber present in broad beans (*Vicia faba*), acting as both modulators of the aryl hydrocarbon receptor (AhRL) involved in autoantigen tolerance and as gut dysbiosis-restoring agents. An interesting hypothesis emerges from the *in-silico* evidence presented by the authors: Metabolites (M-tyramine, taxifolin, Lumarin, and hydroxylated wyerone acid) of certain antioxidant phytochemicals (L-DOPA, quercetin, resveratrol, and wyerone acid) are equally effective ligands of AhRL.

The high-throughput identification of single/multiple food antioxidants associated with a given health benefit is often supported by experimental (animal/cell models) and clinical (humans) evidence. These preliminary steps sometimes prompt the development of nutraceuticals (extracted/purified bioactive with presumed activity) that should be retested for such bioactivities to gain structure-specific evidence (QSAR) on the involved molecular mechanisms, bio-safety, and metabolic impact (7). He et al. evaluated the dose-response (250–2,000 mg.day^−1^) effect of oral Vitamin C supplementation in 98 middle-age (41 ± 8 y, 58% female) patients with non-alcoholic fatty liver disease (NAFLD) participating in a double-blind randomized-clinical trial. After 12 weeks of supplementation, positive effects on glycemic homeostasis, hepatic health, and gut microbiota-liver axis function were observed in a dose-dependent manner. However, the administration of antioxidants has negative effects under certain clinical conditions. Chen et al. observed that lycopene (especially at a high dose) aggravates alcohol-induced gastric injury (local necrosis + shedding epithelial cells + abnormal gastric microstructure) in Kunming mice by an unknown mechanism associated with a presumable alcohol-lycopene interaction but not lycopene alone which exerts its antioxidant or anti-inflammatory effect in absence of alcohol.

The last article included in this Research Topic of Frontiers in Nutrition addressed a relevant microstructural aspect associated with the effective delivery/absorption and *in situ* activity of citrus bioactive compounds. Bruno et al. examined the morphology (dynamic light scattering, transmission electron microscopy), stability (simulated GI environment), absorption/cytotoxicity (Caco-2 cells/monolayer), and bioactivity (anti-inflammatory effect, tight junction-related genes) of extracellular vesicles from the orange (*Citrus sinensis*) juice (CS-EVs). Authors demonstrated that CS-EVs are very stable under simulated GI conditions, are readily absorbed and promote the expression of anti-inflammatory (i.e., ICAM1 and HMOX-1) and tight junctions (i.e., OCLN,CLDN1, and MLCK)-related genes. Even though the chemical nature of the bioactive compounds associated with CS-EVs' positive effects on intestinal integrity is not revealed, the authors hypothesize that some of them have an antioxidant character (e.g., flavonoids), as has been documented for this citrus fruit (8, 9).

## Author Contributions

All authors listed have made a substantial, direct, and intellectual contribution to the work and approved it for publication.

## Conflict of Interest

The authors declare that the research was conducted in the absence of any commercial or financial relationships that could be construed as a potential conflict of interest.

## Publisher's Note

All claims expressed in this article are solely those of the authors and do not necessarily represent those of their affiliated organizations, or those of the publisher, the editors and the reviewers. Any product that may be evaluated in this article, or claim that may be made by its manufacturer, is not guaranteed or endorsed by the publisher.
